# Simulation Training: Evaluating the Instructor’s Contribution to a Wizard of Oz Simulator in Obstetrics and Gynecology Ultrasound Training

**DOI:** 10.2196/mededu.6312

**Published:** 2017-04-21

**Authors:** Aric Katz, Ronnie Tepper, Avraham Shtub

**Affiliations:** ^1^ Technion Industrial Engineering & Management Technion Haifa Israel; ^2^ Simultech Department of Obstetrics & Gynecology Meir Medical Center Kfar-Saba Israel; ^3^ Sackler Faculty of Medicine Tel Aviv University Tel Aviv Israel; ^4^ Technion - Israel Institute of Technology Industrial Engineering & Management Technion Haifa Israel

**Keywords:** distance learning, feedback, simulation training, evaluation research

## Abstract

**Background:**

Workplaces today demand graduates who are prepared with field-specific knowledge, advanced social skills, problem-solving skills, and integration capabilities. Meeting these goals with didactic learning (DL) is becoming increasingly difficult. Enhanced training methods that would better prepare tomorrow’s graduates must be more engaging and game-like, such as feedback based e-learning or simulation-based training, while saving time. Empirical evidence regarding the effectiveness of advanced learning methods is lacking. Objective quantitative research comparing advanced training methods with DL is sparse.

**Objectives:**

This quantitative study assessed the effectiveness of a computerized interactive simulator coupled with an instructor who monitored students’ progress and provided Web-based immediate feedback.

**Methods:**

A low-cost, globally accessible, telemedicine simulator, developed at the Technion—Israel Institute of Technology, Haifa, Israel—was used. A previous study in the field of interventional cardiology, evaluating the efficacy of the simulator to enhanced learning via knowledge exams, presented promising results of average scores varying from 94% after training and 54% before training (n=20) with *P*<.001. Two independent experiments involving obstetrics and gynecology (Ob-Gyn) physicians and senior ultrasound sonographers, with 32 subjects, were conducted using a new interactive concept of the WOZ (Wizard of OZ) simulator platform. The contribution of an instructor to learning outcomes was evaluated by comparing students’ knowledge before and after each interactive instructor-led session as well as after fully automated e-learning in the field of Ob-Gyn. Results from objective knowledge tests were analyzed using hypothesis testing and model fitting.

**Results:**

A significant advantage (*P*=.01) was found in favor of the WOZ training approach. Content type and training audience were not significant.

**Conclusions:**

This study evaluated the contribution of an integrated teaching environment using a computerized interactive simulator, with an instructor providing immediate Web-based immediate feedback to trainees. Involvement of an instructor in the simulation-based training process provided better learning outcomes that varied training content and trainee populations did not affect the overall learning gains.

## Introduction

Medical education is becoming increasingly challenging. Physicians must master an ever-expanding knowledge base; yet, they are constrained by a limited educational time frame. Didactic learning (DL) is no longer sufficient, hence interactive methods are needed. Thus, and in part due to the Internet, an alternative—Web-based educational—content has emerged [1]. Examples include flipped classroom [1,2], simulation-based training [3,4], and e-learning [5,6]. Although some of these methods have demonstrated encouraging results, others are still experimental and require a stronger evidence-based background [7,8]. Instructors still tend to perceive these methods as a demanding effort. Solid empirical evidence regarding the effectiveness of these novel teaching approaches is needed. Current publications lack objective quantitative evidence (knowledge test scores) for comparing various advanced training methods among themselves or with DL [5-7,9].

In addition to increase our knowledge base, cognitive learning can change our beliefs and the way we see and understand events. A major step in understanding the way people learn evolved in the late 1950s when the field of cognitive science emerged [10]. Cognitive science brought with it new experimental tools and methodologies that contributed to empirical and qualitative research. Novel approaches for enhanced teaching are emerging, yet a change in DL approaches has been implemented only minimally in schools. Many researchers believe that didactic teaching fails to prepare students for challenges they are likely to encounter in their professional lives. “Human competencies such as teamwork, cooperation, customer orientation, and entrepreneurial thinking are gaining more and more importance. However, didactic education and training concepts in universities and industries do not fulfill the new requirements” [9]. Moreover, accreditation institutions require graduates to communicate better, resolve engineering problems and be part of a multidisciplinary team [1].

e-learning is a powerful, cost-effective training tool. Although some have described it as boring and monotonous [6] when compared with DL and technology-assisted learning (TAL), it ranked as the most valuable training method [11]. A study that compared DL and TAL found that most participants (61%) preferred to attend the TAL courses. -learning was described as a cost effective, dynamic and interactive training method that brought new expertise to learners and reinforced existing training [11]. Additionally, e-learning is a platform with reusable materials, providing free and distance-learning to rural regions; yet, its effectiveness as a standalone solution is questioned [5]. The efficacy of e-learning is not yet known. One study attempted to evaluate the addition of interactivity to e-learning via interviews and questionnaires, comparing DL, e-learning, and mixed classes (e-learning combined with interactive class work). Students who attended the mixed classes reported the highest satisfaction. They reported that e-learning was more effective than classroom learning, yet it fell short on supplying social and teamwork skills that are relevant to the work environment [5].

To improve medical education and training, we developed a novel, low-cost, low-fidelity, accessible telemedicine simulator at the Technion—Israel Institute of Technology, Haifa, Israel—using a Wizard of Oz (WOZ) simulator. The WOZ is a well-established method for simulating the functionality and user experience in which a human operator, the Wizard, mediates the interaction. Using a human wizard to mimic certain operations of a potential system is particularly useful in situations where extensive engineering effort would otherwise be needed to explore the design possibilities offered by such operations [12].

The WOZ simulator features a remote instructor (the Wizard) in the training loop, controlling students’ learning. This approach enables trainers to effectively detect learners’ flawed mental models (misconceptions) and supply corrective immediate feedback during the training sessions [13]. Web-Based immediate, interactive human feedback provides immense advantages to enhanced learning [3,4,7,14-16]. Unlike traditional e-learning, the WOZ concept incorporates a two-way discussion, with immediate feedback, which can help improve the student’s understanding [14]. Initial results regarding the usability and efficacy of the WOZ simulator in training interventional cardiologists, emergency medicine physicians, and medical students, are promising [3,4]. The WOZ simulator was invented following an unsuccessful attempt to develop a fully automated medical simulator at the Technion. The simulator failed because computers lack human intuition and human-like engagement that acknowledge complex and abstract questions [6]. The WOZ simulator could overcome these issues by returning the instructor (Wizard) to the training loop.

The WOZ simulator is a novel form of a low-fidelity, semiautomatic simulator designed to enhance medical education. It has been used to remotely train physicians in fields of emergency medicine, pediatrics, and interventional cardiology [3,4]. Promising results were noticed when training 20 interventional cardiologists on radiation protection. Knowledge improvement measured via knowledge exams before and after training showed a 40% improvement (94–54) with *P*<.001 [3].

The field of Obstetrics and Gynecology (Ob-Gyn) was chosen for this study due to its focus on knowledge-related tasks and diagnostic skills, such as US imagery interpretation. Fields that rely on knowledge-specific tasks and diagnostic skills better match the remote training, low-fidelity, instructor-led WOZ simulator platform.

## Methods

### Study Design

**Figure 1:**
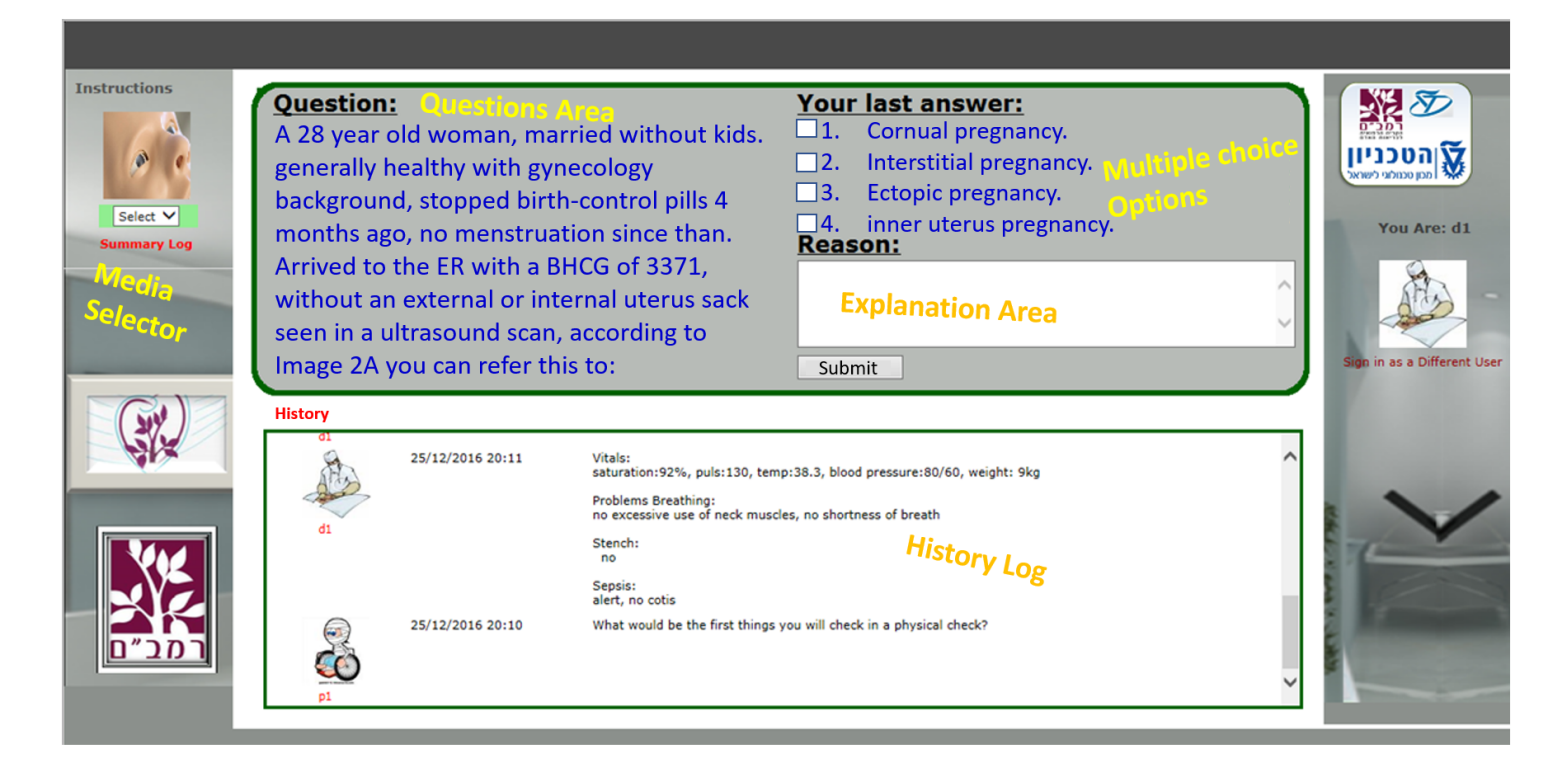
User interface of the simulator—a multiple choice or open question interface.

**Figure 2:**
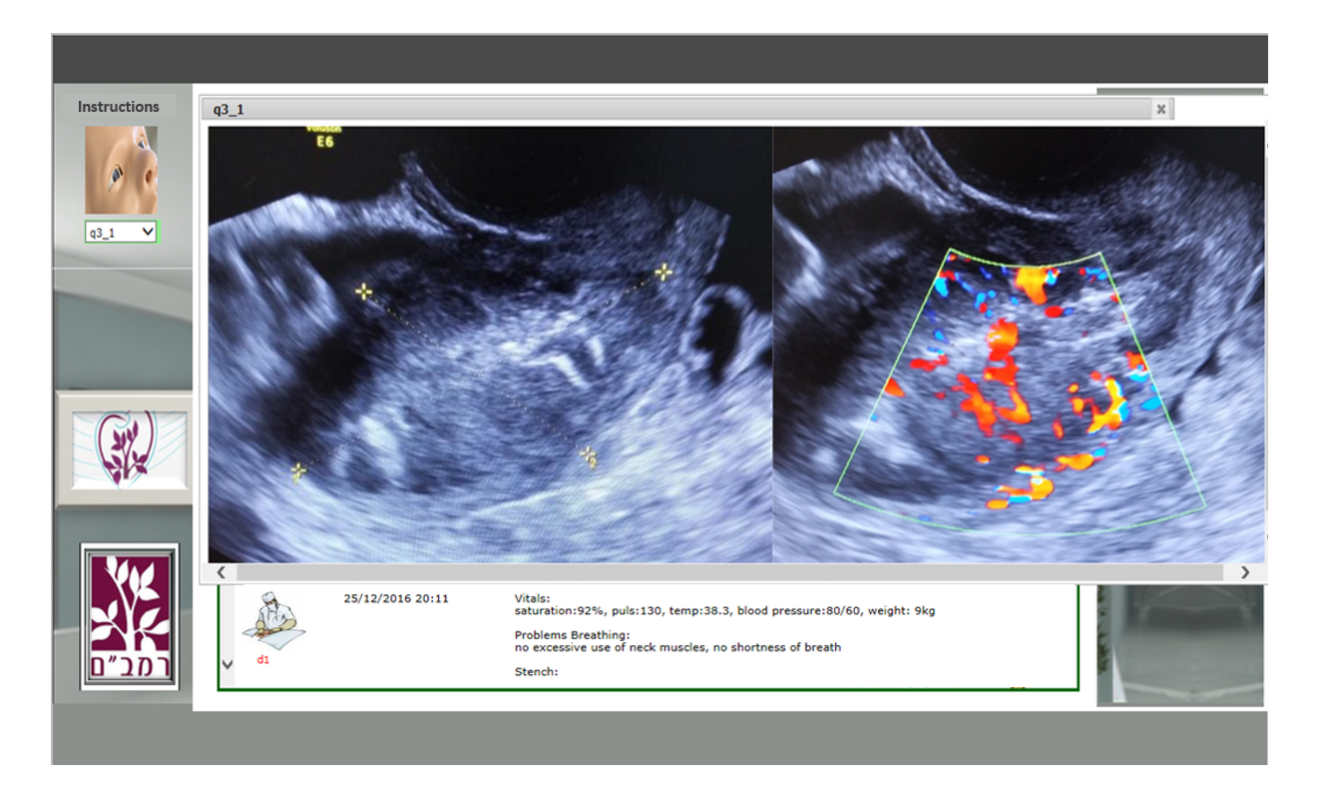
Simulator media display. Selecting a media option from the media drop-down displays a pop-up with the relevant image, lab results, or video.

This prospective study was performed at the Simultech Center for Simulation in Medicine. The Center specializes in Ob-Gyn training. The WOZ simulator was accessed through a weblink (Figure 1). Training started by clicking the image of the patient in the upper left corner, which resulted in a pop-up displaying the patient’s history and relevant case data. The first question appeared in the questions area when the pop-up was closed. Questions were open-ended or multiple-choice, in which case a list of possible answers was presented. The questions could include supporting media, such as images, videos, documents, or presentations (Figure 2). Open questions required a written explanation and multiple-choice questions required choosing the correct answer from the available options and clicking on the submit button.

In the automated e-learning mode, the follow-up question would immediately appear. In the WOZ mode, the instructor could proceed to the follow-up question or ask additional questions, send clarifying information, or skip some questions depending on the trainee’s progress. After answering the final question, automated feedback would be presented to the trainee in the e-learning format, whereas in the WOZ format, the instructor would provide feedback in an open conversation.

### Trainees

The design of our experiment required that there be a number of medical trainees sharing a similar medical knowledge background as well as qualified medical trainers. The Simultech Simulation Center satisfied these requirements. Integrating our WOZ simulator experiments into Simultech’s training schedule was done in two different courses and included a total of 32 Ob-Gyn ultrasonography specialists.

### Population

The first experiment group consisted of 18 (12 men and 6 women) physicians who were participating in an Ob-Gyn ultrasound imaging fellowship program. The second experiment group included 14 women, who were senior ultrasound technicians with 5-20 years of experience.

### Intervention Group

In the first experiment group, 8 random subjects went through the WOZ training session, whereas in the second experiment group 7 subjects (4 from the ovary subgroup and 3 from the uterus subgroup) were trained using the WOZ format. The interactive WOZ training session used the same case as the e-learning session, yet subjects received interactive, remote (sitting in a different room) Web-based immediate feedback from an instructor monitoring their progress (Figure 4). Case progress was controlled by the instructor based on expertise displayed by the student. Additionally, a final frontal feedback was presented and a posttraining knowledge exam was conducted.

**Figure 3:**
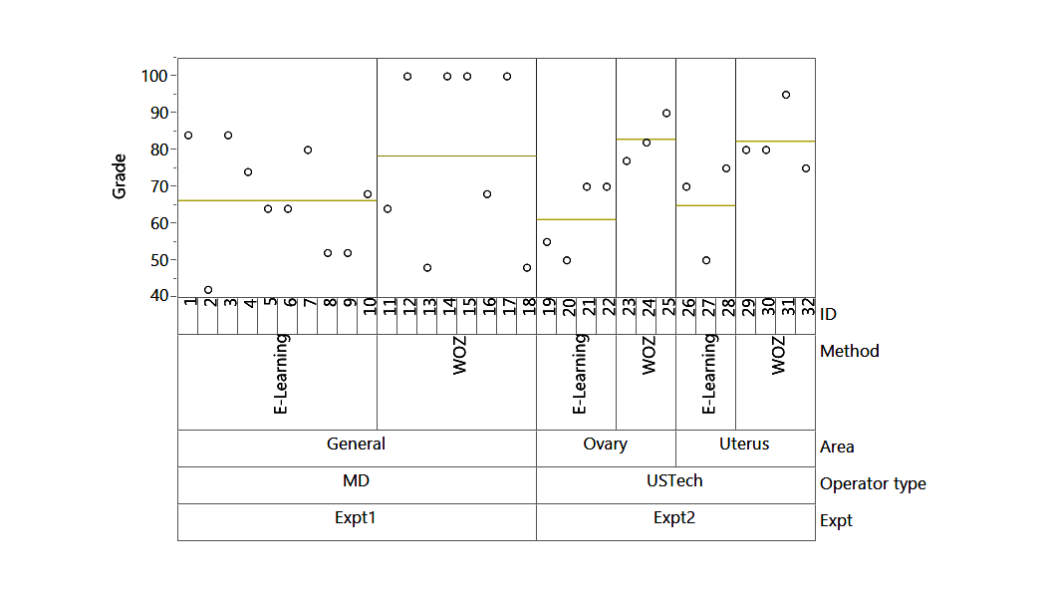
Variability chart presenting the difference between block experiments 1 and 2 with medical subject area, and WOZ and e-learning.

### Control Group

In the first experiment group, 10 random subjects went through the e-learning training session, whereas in the second experiment group, 7 subjects (3 from the ovary subgroup and 4 from the uterus subgroup) were trained using the e-learning format. e-learning sessions included self-paced training on the simulator and a post-session knowledge test. The self-paced training (a new question appeared immediately after the previous question was answered) included a set of questions, their supporting media and a self-assessment feedback table (questions, trainee’s answers, and expected answers with detailed explanations) received at the end of the session. Case questions, their detailed answers and the knowledge test were developed and validated by Simultech instructors and the medical professionals supporting them. The validated medical case was uploaded to a local server, accessed by a local network at Simultech.

### Materials

All evaluators were tested by a post-training, objective knowledge exam. All knowledge exams (1 for each of the 3 knowledge areas: general Ob-Gyn, uterus, and ovary) were validated by medical professionals from the Simultech Simulation Center and included 10 open questions that were evaluated and scored by professional medical supervisors.

Control groups (e-learning) from both experiments received an automated, self-assessment computerized case, where trainees answered a set of questions (Figure 1). Each e-learning session was followed by automated feedback, which presented trainees with the case questions, their answers, and the correct answers with detailed explanations. The intervention group (WOZ) received the same computerized case accompanied by a human trainer supplying Web-based immediate feedback and clarifications for each question and a final frontal debriefing at the end of each session.

### Outcome Measure

The outcome measure evaluated in both experiments was knowledge gain based on the training received. Knowledge gain was evaluated using knowledge exams that were given to all subjects upon completion of their computerized training. According to the magnitude of difference between knowledge exam scores, the significance of instructors’ contribution to the learning process can be deduced.

### Training Development and Teaching

Before all training sessions, students were informed that the training was part of a research project. Due to a current change in Simultech’s policy toward minimizing e-learning trainings, the experiment was divided into two sections: Training of Ob-Gyn physicians as part of a full-day training event and training senior US technicians as part of a continuing education program.

Training sessions for the first experiment (Ob-Gyn physicians) were conducted by professional instructors (Simultech’s instructor team). They focused on supplying new techniques, knowledge reinforcement, and skill acquisition. Simultech’s instructors are certified teachers with no medical background. Instructors study specific medical cases built by Simultech’s medical professionals. The WOZ simulator training was scheduled to run once a week or every 2 weeks, depending on instructors’ availability. Participants were randomly chosen to use the e-learning mode (control group) or the interactive WOZ mode (intervention group).

The second experiment included 14 of 23 (female) senior ultrasound technicians who attended a senior technicians’ ultrasound course. The course included four meetings in 1 month. The course was attended by 23 senior ultrasound technicians (only 14 participated in the final experiment). A month after the course ended, a half-day training was added for students to practice and train with the WOZ simulator on cases designed by students during the course. Contrary to previous ultrasound senior technician courses that used DL during class time, this course included a homework assignment (building medical training case’s questions). The homework required students to study a specific medical topic, whereas some of the class time was used to train, instruct, and facilitate team learning, using Simultech instructors as medical consultants.

The WOZ simulator and the new exercise were presented to the ultrasound technicians at the first-class meeting. Students were divided into a uterus and an ovary subgroup and were asked to build a training case for the WOZ simulator that would include the following:

- A minimum of 10 training knowledge questions (multiple choice and open questions);
- Media to support the questions (images, video, lab results, patient’s background);
- Detailed best practice answers with additional supporting media;
- A set of 10 open questions for an objective knowledge exam to be administered to each group after simulator training.

Additionally, students were informed that they would assume the role of instructors while training their peers on the WOZ simulator using their prebuilt training case. During the course, each student was responsible for developing one training question, its answer, and all supporting media, per his or her assigned topic of uterus or ovary. This assignment required each student to study a specific topic using written information, consult with the course staff and his or her medical coworkers. A team leader was chosen for each group to integrate all the questions into one training case that was validated by the Simultech training staff. Each group wrote a knowledge test of 10 open questions for each case. Several selected topics (questions built by students) that were not integrated into the simulator cases were presented as lectures at the end of the fourth meeting of the course.

**Figure 4:**
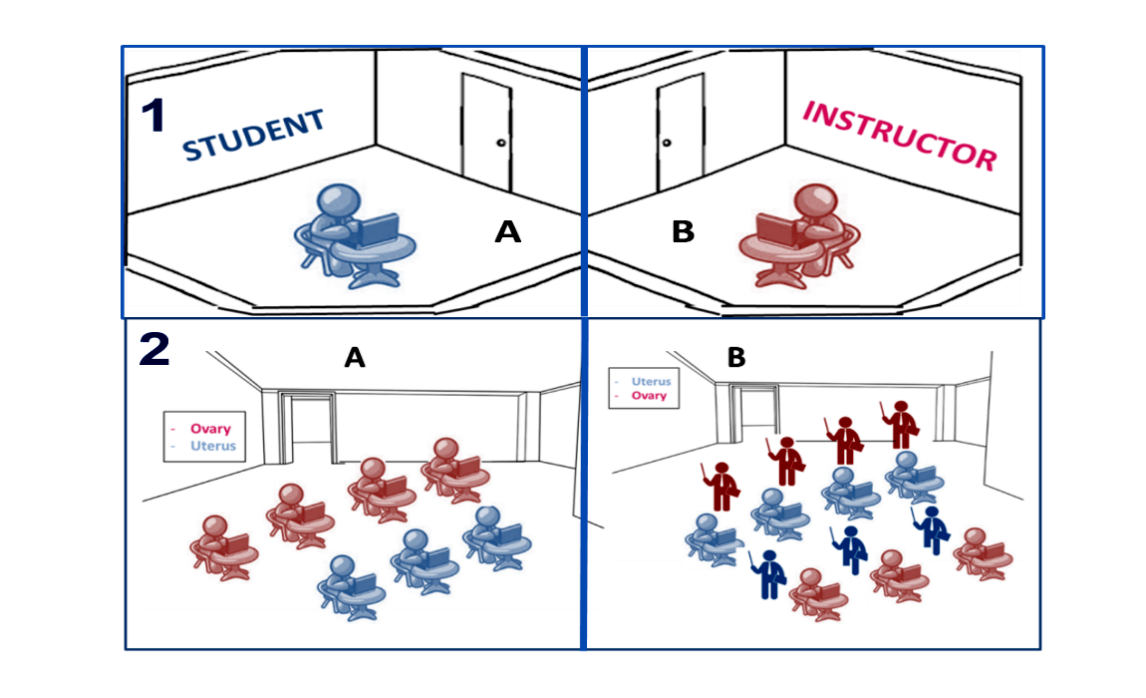
(1:A) E-learning training mode—a fully automated case. The user interacts solely with a computer. (1:A+B). WOZ training mode: Students interact with a remote instructor receiving Web-based immediate feedback and support. (2:A) E-learning training class: students train on the simulator with no instructor support. (2:B) WOZ training class: students train on the simulator with an instructor (from the opposite group) leading, supporting and supplying Web-based immediate feedback during training.

The second experiment was held at Simultech a month after the first course ended. Fourteen students participated in the final simulator training (7 from each group). As illustrated in Figure 4, the training began with the e-learning session (control group), where 4 students from the uterus group trained on the ovary case and 3 students from the ovary group trained on the uterus case. After the first group of students finished their self-paced, self-assessed training, they received a written, open question, by topic (uterus or ovary), and a knowledge exam regarding the case they just completed. The second stage of the experiment included interactive WOZ training (intervention group) for the remaining 7 students, where 3 from the uterus group trained on the ovary case and 4 from the ovary group trained on the uterus case. This session included all 14 students, as the 7 students from the first stage instructed the 7 students from the second stage. Students undertaking the role of instructors assisted their classmates by clarifying questions and supplying Web-based immediate feedback. The same written knowledge tests (uterus and ovary) were given to the (WOZ and e-learning) subgroups after they completed the training session. Exams were evaluated and graded by an Ob-Gyn who guided the course from Clalit Healthcare Services.

### Using the Simulator

The simulator used for training by the two groups, is a low-fidelity, Web-based application that was developed using Microsoft SharePoint 2010 technology. The simulator was used in previous studies and its efficacy as a training tool in various fields of medicine was evaluated [3,4]. For this study, minor changes were made to the simulator’s user interface to enable a more e-learning like look and feel (Figure 1). The main advantage of using this platform was its dual mode support that can run the same case in fully automated e-learning mode and in interactive WOZ mode. The WOZ mode enabled the trainer to control a student’s progress and supply Web-based immediate feedback to correct any misconceptions or errors [13].

### Statistical Analysis

The JMP statistical package was used to compare the results of the knowledge tests taken after the completion of each training mode: e-learning and the interactive WOZ. The results for both experiments of 32 participants are displayed in Figure 3. A statistical model with the factors such as operator type (physicians/US senior technicians), method (WOZ/e-learning), and area (general, ovary, uterus) nested in “operator type” was fitted for both experiments together. Additionally, an equal variance exam was used to validate that both merged groups (operator type) shared the same variance (Tables 1 and 2). The level of statistical significance was set at 5%.

**Table 1:** Test for equal variance comparing both training experiments (difference of means).

Level			Mean absolute difference	Mean absolute difference
	Count	Standard deviation	Mean	Median
MD_General	18	19.65453	16.41975	16
Ultra sound physicians	14	13.54297	10.2449	9.92857

**Table 2:** Results by tests for equal variance performed.

Test	*F* ratio	Degrees of freedom	Degrees of freedom of denominator	*P* value
O'Brien (.5)	3.2068	1	30	.08
Brown-Forsythe	2.6479	1	30	.11
Levene	3.4262	1	30	.07
Bartlett	1.8723	1		.17
*F* Test 2-sides	2.1062	17	13	.18

## Results

### Evaluators

The first experiment included 18 medical, fellowship physicians who were randomly divided into two subgroups. The first subgroup included 10 e-learning trainees who scored an average of 64% (SD 13), and the second subgroup included 8 interactive (trainer-led) WOZ trainees who scored an average of 79% (SD 24).

The second experiment included 14 females, senior ultrasound technicians. They were randomly divided into 7 e-learning trainees who scored an average of 63% (SD 11) and 7 interactive trainees who scored an average of 83% (SD 7). Each training method was also subdivided based on the selected topic (uterus, ovary). The topic and its interaction with the training method were not significant relative to the grades scored.

### Training Development and Teaching

The first sessions included Simultech instructors training Ob-Gyn physicians as part of a full-day training at Simultech. Trainees were randomly selected to perform the e-learning or the instructor-led WOZ session. The WOZ training included summarized verbal feedback at the end of the interactive case. Most of the WOZ training was done by one dedicated instructor. The knowledge test included general Ob-Gyn and US-related questions taken from previous cases built by Simultech’s professional staff. This part of the training included 18 sessions, spanning more than 6 months.

The second training session lasted 1 day and took place a month after the four meetings of the course. Both training cases were built by course students, supported by Simultech’s medical professionals and were uploaded to the WOZ Web server at the Technion. Training cases and their knowledge tests were divided into 2 knowledge areas (ovary and uterus). The WOZ training for all 7 trainees occurred in the same room with a trainer from the other group sitting behind each trainee. A knowledge exam consisting of 10 open questions was given after the computerized training and scored by an Ob-Gyn, physician an hour after all exams were submitted.

Training sessions lasted from 40 to 60 minutes. All participating students in both experiments completed 10 open questions in a written knowledge exam post-simulator training. All interactive WOZ sessions included 1 instructor training 1 trainee; yet, in previous studies we showed that 1 trainer can train 2 trainees or a small team [4].

### Statistical Analysis

Before running the full model based on both experiments, a homogeneity of variance test was conducted (Tables 1 and 2). Results from this analysis validated that both experiments share the same variance.

A full model with the factors—operator type, method, area nested in operator—was fitted. All individual factors and interactions between method and operator type and between method and area were analyzed using the JMP statistical tool. A total of 32 observations were used from both experiments together (Figure 3 and Table 3). Results from this analysis showed that only the training method was significant with a *P* value of .01 (Table 3). Operator type (Physician or US technician) and content were both found to be insignificant in explaining the variance in exam scores.

**Table 3:** Analysis of variance table with statistical tests for the entire model.

Source	Nparm	DF	Sum of Squares	*F* Ratio	*P* value
Operator type	1	1	1.8399	0.0069	.93
Area (Operator type)	1	1	9.0536	0.0341	.86
Method	1	1	1948.0173	7.3445	.01^a^
Operator type X Method	1	1	109.598	0.4132	.53
Area X Method (Operator type)	1	1	15.4821	0.0584	.81

^a^significant at *P*<.05.

## Discussion

### Principal Findings

This study quantitatively evaluated the contribution of an instructor supplying Web-based immediate feedback to individuals using a computerized, interactive WOZ simulator. Previous research noted the lack of empirical and quantitative studies evaluating the efficacy of simulation-based training in shifting knowledge from opinion based to evidence based [7,8,16]. The addition of an instructor to learning outcomes was evaluated by comparing increases in subjects’ knowledge after using a fully automated, e-learning case study and an interactive, instructor-led case study run on the same platform (WOZ simulator). Results indicated significant added value from the instructor’s contribution, controlling learners’ training progress and supplying Web-based immediate feedback.

Two experiments were conducted to evaluate the contribution of an instructor in a computerized simulated learning environment. They compared an interactive WOZ mode with a fully automated, e-learning mode. Research has revealed that e-learning should become more interactive to achieve better learning [5]. The first experiment tested a group of Ob-Gyn physicians. The second experiment evaluated senior, female ultrasound technicians. We used a cross-over training design (e-learning/instructor led) of the WOZ simulator to evaluate knowledge gained. Training included computer-based practice with (WOZ) and without an instructor (e-learning) and a knowledge test to evaluate training efficiency. We found that training supervised by an instructor who supplied immediate Web-based feedback increased learning outcomes (Table 3). The instructor helped trainees to understand the information better by clarifying Web-based, emphasizing relevant information, and resolving their errors and misconceptions [13].

The experiments were integrated to evaluate the overall contribution of the instructor (man in the loop) to the training process. The joint analysis indicated that the interactive WOZ training presented a significant advantage (*P* values of the method parameter =.0118) compared with the e-learning alternative. The impact of the training method on test grades was reinforced by the lack of statistical significance of the medical subject (area) and its interaction with the training method. Moreover, the experiment factor (operator type) and its interaction with the training method were not significant. This indicates that most of the variability in students’ grades was due to the training method (WOZ vs e-learning).

In addition, a new teaching exercise of enhanced learning was added to the second experiment. The enhanced learning assignment required students (14 ultrasound technicians) to research a specific topic (uterus or ovary), build a WOZ simulator training case based on their research, and assume the role of the instructor and train their fellow classmates.

This exercise had very positive outcomes. Students and the management team described it as the most educational course Simultech had ever provided. Students training on the simulator described their experience as fun and educational, as was previously described by trainees using the WOZ simulator [3,4]. During the interactive WOZ sessions, students confronted their instructors (fellow classmates) on the quality of the case question and supplied feedback on the quality of the supporting media and the clarity of the question. This generated new discussions among students, and the management team was called to help sort out differences of opinion among the students. Furthermore, students and the management team mentioned that this exercise contributed dramatically to team building, increased motivation, and generated new working relations between technicians from different organizations.

### Limitations

The study presented interesting insights regarding the contribution of instructors to a computerized training process, although there were several limitations. The study was conducted with limited access to trainees’ personal and background data. It had a small sample size because ongoing courses at Simultech are constrained to a short timeframe with little flexibility to apply additional content. This study can contribute to the developing field of enhanced learning and can support continued research in this area.

The added value of Web-based immediate feedback and integrating an instructor to simulation-based training were introduced in the literature review [3,4,7,14,16] and in this study; yet training costs increase as well. Simultech’s approach to reduce this overhead includes using trained instructors with no medical background who are directed by medical professionals. All training materials are developed by physicians. Additionally, students were responsible for building their own training and self-instruction units.

### Conclusions

We conducted two independent experiments using a WOZ simulator to evaluate *quantitatively* the contribution of an instructor to learning with a computer-based WOZ simulator training. The results indicate the WOZ training was superior to automated e-learning. In one experiment, students were responsible for developing training materials and for training their peers. Researching and developing an educational unit contributed immensely to the overall student satisfaction. Additionally, this exercise initiated new discussions among students, improved team building, increased motivation, and created new work relations between technicians from various organizations. The results of this study present interesting insights in favor of interactive, instructor WOZ simulator training as compared with automated e-learning.

## Abbreviations

DL: didactic learning
Ob-Gyn: Obstetrics and gynecology
TAL: technology-assisted learning
WOZ: Wizard of Oz

## References

[1] Bishop J, Verleger M (2013). The flipped classroom: a survey of the research. The American Society for Engineering Education.

[2] Prober CG, Heath C (2012). Lecture halls without lectures--a proposal for medical education. N Engl J Med.

[3] Katz A, Shtub A, Solomonica A, Poliakov A, Roguin A (2017). Simulator training to minimize ionizing radiation exposure in the catheterization laboratory. Int J Cardiovasc Imaging.

[4] Katz A, Basis F, Shtub A (2014). Using Wizard of Oz technology for telemedicine. Health Syst.

[5] Alzahrani JG, Ghinea G (2012). Evaluating the impact of interactivity issues on e-learning effectiveness. http://www.ithet.boun.edu.tr.

[6] Sungyoung L, Sora L (2014). Considerations on gamification of e-learning application: case study with phrase building training application. https://www.computer.org/csdl/proceedings/mulgrab/2014/7763/00/index.html.

[7] Issenberg S, McGaghie W, Petrusa E, Lee GD, Scalese R (2005). Features and uses of high-fidelity medical simulations that lead to effective learning: a BEME systematic review. Med Teach.

[8] Wenk M, Waurick R, Schotes D, Wenk M, Gerdes C, Van Aken HK, Pöpping DM (2009). Simulation-based medical education is no better than problem-based discussions and induces misjudgment in self-assessment. Adv Health Sci Educ Theory Pract.

[9] Hoheisel J, Weber F, Windhoff G, Riis JO, Smeds R, Van Landeghem R (2000). New approaches for training education of engineers by using simulation games. Games in Operations Management.

[10] Bransford J, Brown A, Cocking R, Council Nr (2000). How People Learn: Brain, Mind, Experience, and School.

[11] Raghavendra N, Rajini R (2012). A qualified analysis of traditional and technology assisted learning - an IT industry outlook. http://ieeexplore.ieee.org/document/6306703/.

[12] Prommer T, Holzapfel H, Waibel A (2006). Rapid simulation-driven reinforcement learning of multimodal dialog strategies in human-robot interaction. https://www.researchgate.net/publication/221478221_Rapid_simulation-driven_reinforcement_learning_of_multimodal_dialog_strategies_in_human-robot_interaction.

[13] Klein G, Baxter H (2009). Semantic Scholar.

[14] Orsmonda P, Maw S, Park J, Gomez S, Crook A (2011). Moving feedback forward: theory to practice. Assess Eval High Educ.

[15] Warren JN, Luctkar-Flude M, Godfrey C, Lukewich J (2016). A systematic review of the effectiveness of simulation-based education on satisfaction and learning outcomes in nurse practitioner programs. Nurse Educ Today.

[16] Salas E, Wildman J, Piccolo R (2009). Using simulation-based training to enhance management education. ‎Acad Manag Learn Edu.

